# A Structural Equation Analysis of Body Image, Stigma, Depression, and Resilience Among Active-Duty Female Military Service Members

**DOI:** 10.5888/pcd23.260225

**Published:** 2026-10-22

**Authors:** Reneé Z. Banks

**Affiliations:** 1 Trident University International, College of Health and Human Services, Chandler, Arizona

## Abstract

Body image dissatisfaction and weight stigma may affect psychological readiness for duty among female service members, but evidence for active-duty populations is limited. Cross-sectional data were collected via validated self-report instruments from 193 active-duty female US service members in 2025. Partial least squares structural equation modeling was used to analyze data. Body image dissatisfaction was associated with lower resilience (β = −0.388) and greater weight stigma (β = −0.587). Weight stigma was not associated with resilience or depression and did not mediate outcomes. Emotional regulation moderated the relationship between weight stigma and resilience, suggesting that the military’s approach to health promotion may be strengthened by incorporating evidence-based strategies for enhancing body image satisfaction, such as emotional regulation and resilience-building techniques.

**Summary**

**What is already known on this topic?**

Weight stigma is associated with depression, reduced self-esteem, and maladaptive coping in US civilian populations, but less is known about its effects among active-duty female US service members.

**What is added by this report?**

Among active-duty female service members, body image dissatisfaction was associated with greater weight stigma and lower resilience, whereas weight stigma was not associated with resilience or depression. Emotional regulation moderated the relationship between weight stigma and resilience.

**What are the implications for public health practice?**

Military health programs should address body image–related stressors, use stigma-aware communication, and integrate emotional regulation and resilience-building strategies to support psychological readiness for duty.

## Objective

Weight stigma is the social devaluation of people because of body weight and may become internalized as negative self-beliefs. Prior research links weight stigma and weight bias internalization with depression, reduced self-esteem, maladaptive coping, stress, and other adverse health outcomes in civilian populations (1–4). Body image dissatisfaction is also associated with psychological distress and reduced quality of life (5). Female service members experience body image dissatisfaction and weight stigma in a military context in which body composition, appearance, and fitness standards are connected to readiness expectations and professional identity (6). This study examined associations among body image dissatisfaction, weight stigma, resilience, and depression among active-duty female US military service members. Resilience was conceptualized as the capacity to adapt to stress and maintain psychological functioning. The study also assessed whether emotional regulation and self-compassion moderated associations between weight stigma and psychological outcomes.

## Methods

A quantitative cross-sectional study was conducted using an anonymous Qualtrics survey. Participants were recruited from April 18 through May 9, 2025, through military-affiliated Facebook groups for women’s mentorship and human resources professionals and professional networks accessible to active-duty women across duty locations, including those stationed in the US and abroad. Eligibility screening required participants to self-report being female, aged 18 years or older, and currently serving on active duty in the US military. Military status was not independently verified through personnel records. Of 234 respondents, 193 met eligibility and data-quality criteria and were included in analysis (participation rate, 82.5%).

All variables were self-reported, including height and weight, which were used to calculate body mass index (BMI) as weight in kilograms divided by height in meters squared. BMI was categorized as underweight (BMI <18.5), normal weight (BMI 18.5–24.9), overweight (BMI 25.0–29.9), and obese (BMI ≥30.0). 

Body image dissatisfaction was measured by the 23-item Body–Esteem Scale for Adolescents and Adults (7); weight stigma by the 12-item Weight Self-Stigma Questionnaire (8); resilience by the 10-item Connor–Davidson Resilience Scale (9); depression by the 9-item Patient Health Questionnaire-9 (10); emotional regulation difficulties by the 16-item Difficulties in Emotion Regulation Scale-16 (11); and self-compassion by the 12-item Self-Compassion Scale-Short Form (12). Higher DERS-16 scores indicate greater difficulty regulating emotions; therefore, lower scores reflect stronger emotional regulation abilities. Missing data were minimal (3.4%) and handled using mean imputation.

Partial least squares structural equation modeling in SmartPLS 4 (SmartPLS GmbH) tested direct, indirect, and moderating relationships. Statistical significance was evaluated at α = .05 using 2-tailed tests with 5,000 bootstrap resamples. For each path, the standardized coefficient (β), bootstrapped *t* statistic, and corresponding *P* value were reported. Measurement model assessment included internal consistency reliability, convergent validity, and discriminant validity. Approval to conduct this study was obtained from the institutional review board at Trident University International. All procedures involving human participants were conducted in accordance with applicable ethical standards. Informed consent was obtained electronically before survey participation.

## Results

The final sample included 193 active-duty female service members aged 18 to 60 years; most were aged 30 to 44 years (62.2%). Participants represented multiple rank and service-length categories (Table 1). On the basis of self-reported height and weight, 43.5% of participants were classified as overweight and 31.6% as obese.

**Table 1:** Characteristics of Participants (N = 193) in a Survey About Body Image Dissatisfaction and Weight Stigma Among Active-Duty Female Service Members, US, 2025

Characteristic	No. (%)^a^
**Age, y**	
18–29	67 (34.7)
30–44	120 (62.2)
45–60	6 (3.1)
**BMI category^b^**	
Underweight (<18.5)	1 (0.5)
Normal weight (18.5–24.9)	47 (24.4)
Overweight (25.0–29.9)	84 (43.5)
Obese (≥30.0)	61 (31.6)
**Military rank**	
Junior enlisted	19 (9.8)
Senior enlisted	74 (38.3)
Junior officer	45 (23.3)
Senior officer	47 (24.4)
Warrant officer (CW2–CW5)	6 (3.1)
Missing/not reported	2 (1.0)
**Years of service**	
Junior (0–4)	75 (38.9)
Mid (5–10)	66 (34.2)
Senior (≥11)	52 (26.9)
**Race and ethnicity^c^**	
Black or African American	54 (28.0)
Hispanic	19 (9.8)
White	109 (56.5)
Other	11 (5.7)

Abbreviation: BMI, body mass index.

a Percentages may not total 100 because of rounding.

b BMI was based on self-reported height and weight and calculated as weight in kilogram divided by height in meters squared.

c Race and ethnicity categories shown are the response options presented in the survey. “Other” includes Asian, Native American, Pacific Islander, and multiracial.

For each structural path, β represents the standardized path coefficient, and the reported *P* value corresponds to the bootstrapped *t* statistic testing whether the coefficient differed significantly from zero. Body image dissatisfaction was significantly associated with lower resilience (β = −0.388; *t* = 6.27; *P* < .001) and greater weight stigma (β = −0.587; *t* = 14.27, *P* < .001) but was not significantly associated with depression (β = −0.107; *t* = 1.46; *P* = .14) (Table 2). Weight stigma was not significantly associated with resilience (β = 0.018; *t* = 0.25; *P* = .80) or depression (β = 0.057; *t* = 0.84; *P* = .40). The model explained 50.2% of variance in resilience, 42.9% in depression, and 34.5% in weight stigma.

**Table 2:** Structural Model Results and Hypothesis Testing for a Study About Body Image Dissatisfaction and Weight Stigma Among Active-Duty Female Service Members (N = 193), US, 2025

Hypothesis/path^a^	β^b^	*t*	*P* value^c^	95% CI for indirect effect^d^	Hypothesis supported
**Direct effects**					
Body image dissatisfaction to resilience	−0.388	6.27	<.001	—	Yes
Body image dissatisfaction to depression	−0.107	1.46	.14	—	No
Body image dissatisfaction to weight stigma	−0.587	14.27	<.001	—	Yes
Weight stigma to resilience	0.018	0.25	.80	—	No
Weight stigma to depression	0.057	0.84	.40	—	No
**Indirect effects**					
Body image dissatisfaction to weight stigma to resilience	−0.087	1.90	.06	−0.173 to 0.006	No
Body image dissatisfaction to weight stigma to depression	−0.026	0.55	.58	−0.121 to 0.069	No
**Interactions**					
Interaction of weight stigma and emotional regulation on resilience	0.150	2.00	.046	—	Yes
Interaction of weight stigma and self-compassion on resilience	0.147	1.91	.06	—	No

Abbreviation: —, does not apply.

a Direction of effect should be interpreted in relation to the scoring of each measure. The body image construct was measured with the Body Esteem Scale for Adolescents and Adults (7); lower body-esteem scores indicate greater body image dissatisfaction. Thus, the negative coefficient for body image dissatisfaction to resilience indicates that greater body image dissatisfaction was associated with lower resilience, and the negative coefficient for body image dissatisfaction to weight stigma indicates that greater body image dissatisfaction was associated with greater weight stigma.

b β = standardized path coefficient.

c *P* values correspond to 2-tailed bootstrapped *t* statistics testing whether each standardized path coefficient differed from zero; statistical significance was evaluated at α = .05.

d Shown only for indirect effects; they were the bootstrapped 95% CIs available from the mediation analysis.

Mediation analysis did not support weight stigma as an indirect pathway between body image dissatisfaction and outcomes. The indirect effect on resilience was not significant (β = −0.087 [95% CI, −0.173 to 0.006]; *t* = 1.90; *P* = .06) because the 95% CI included zero. Similarly, the indirect effect on depression was not significant (β = −0.026 [95% CI, −0.121 to 0.069]; *t* = 0.55; *P* = .58). Emotional regulation significantly moderated the relationship between weight stigma and resilience (β = 0.150; *t* = 2.00; *P* = .046). In practical terms, the negative association between weight stigma and resilience was weaker among participants reporting stronger emotional regulation abilities. Self-compassion showed a nonsignificant moderating effect on resilience (β = 0.147; *t* = 1.91; *P* = .06); other interaction effects were not significant.

## Discussion

This study adds to limited public health evidence on body image, weight stigma, and psychological readiness among active-duty female service members. Body image dissatisfaction was associated with lower resilience, suggesting that negative body-related self-perceptions may be relevant to coping capacity in military settings. The absence of a direct association of body image dissatisfaction with depression suggests that depressive symptoms may reflect more complex pathways involving social support, occupational stress, unit cohesion, prior mental health history, or other unmeasured factors.

Prior research on civilian populations has linked weight stigma with depression, reduced self-esteem, maladaptive coping, stress, and other adverse health outcomes (1–4). In this active-duty female military sample, however, weight stigma was not significantly associated with resilience or depression and did not mediate relationships between body image dissatisfaction and psychological outcomes. These findings should be interpreted cautiously. Measurement issues, sample characteristics, restricted variability, military culture, or unmeasured military contextual factors may have contributed to nonsignificant findings. The study did not measure unit cohesion, leadership climate, branch, occupational specialty, or exposure to formal resilience training; future research should include these contextual variables.

The BMI distribution also warrants caution. Most participants were classified as overweight or obese based on BMI values, but BMI based on self-reported height and weight may be of especially limited use in military populations because it does not capture muscularity, body composition, physical performance, or service-specific standards of body composition. The BMI distribution may also reflect self-selection among participants with greater interest in weight- or body-related issues rather than the active-duty female force overall. 

Emotional regulation was the most actionable finding. Participants with stronger emotional regulation abilities appeared better able to maintain resilience despite weight stigma. Military health promotion programs could integrate evidence-based strategies such as mindfulness-based stress reduction, cognitive-behavioral stress management, and dialectical behavior therapy-informed emotional regulation skills into resilience training, behavioral health services, and leadership development. These programs could be tailored to promote skills in stigma-aware communication and to address body image concerns and the gendered pressures female service members may experience when military standards of body composition intersect with broader societal ideals.

This study has limitations. The cross-sectional design precludes causal inference, and all data were self-reported, introducing potential response bias, recall bias, and common method bias. Convenience and snowball sampling through online networks may have introduced self-selection bias and limited representativeness. The sample was restricted to active-duty female service members, limiting generalizability to male service members, Reserve and National Guard personnel, veterans, and civilians. Subgroup imbalances by BMI and rank may also have affected interpretation of data on subgroups.

Body image dissatisfaction was associated with lower resilience among active-duty female service members, whereas weight stigma was not a significant predictor of resilience or depression in this sample. Military health promotion initiatives should move beyond promoting the physical metrics of readiness and incorporate body image–informed, stigma-aware, and emotional regulation–focused strategies to support resilience and readiness.
